# Immune-Mediated Cerebellar Ataxia Associated With Neuronal Surface Antibodies

**DOI:** 10.3389/fimmu.2022.813926

**Published:** 2022-02-17

**Authors:** Yu Jia, Mingyu Li, Dawei Li, Mengyao Zhang, Huifang Wang, Lidong Jiao, Zhaoyang Huang, Jing Ye, Aihua Liu, Yuping Wang

**Affiliations:** ^1^ Department of Neurology, Xuanwu Hospital, Capital Medical University, Beijing, China; ^2^ Beijing Key Laboratory of Neuromodulation, Capital Medical University, Beijing, China; ^3^ Center of Epilepsy, Beijing Institute for Brain Disorders, Capital Medical University, Ministry of Science and Technology, Beijing, China; ^4^ Institute of Sleep and Consciousness Disorders, Beijing Institute of Brain Disorders, Collaborative Innovation Center for Brain Disorders, Capital Medical University, Beijing, China

**Keywords:** immune-mediated cerebellar ataxia, neuronal surface antibodies, CSF analysis, oligoclonal bands, immunotherapy

## Abstract

**Background:**

Immune-mediated cerebellar ataxias (IMCAs) are common in paraneoplastic cerebellar degeneration (PCD) but rarely occur in patients with neuronal surface antibodies (NSAbs). Although cerebellar ataxias (CAs) associated with anti-NMDAR and anti-CASPR2 have been reported in a few cases, they have never been studied systematically. This study aimed to analyze the characteristics of anti-NSAbs-associated CAs.

**Methods:**

A retrospective investigation was conducted to identify patients using the keywords *IMCAs* and *NSAbs*. We collected the clinical data of 14 patients diagnosed with anti-NSAbs-associated CAs.

**Results:**

The median age was 33 years (16-66), and the male-to-female ratio was 4:3. Nine were positive for NMDAR-Ab, two for LGI1-Ab, two for CASPR2-Ab, and one for AMPA2R-Ab. CAs were initial symptoms in three patients and presented during the first two months of the disease course (10 days on average) among the rest of the patients. After the immunotherapy, two cases were free from symptoms, and eight cases recovered satisfactorily (10/14, 71.4%). Compared with other causes of IMCAs, anti-NSAbs were more frequently associated with additional extra-cerebellar symptoms (85.7%), mostly seizures (78.6%) and mental abnormalities (64.3%). In the CSF analysis, pleocytosis was detected in ten patients (71.4%) and oligoclonal bands (OB) were observed in nine patients (64.3%). Moreover, compared with PCD and anti-GAD65-Ab-associated CAs, anti-NSAbs-associated CAs showed a better response to immunotherapy.

**Conclusion:**

IMCAs are rare and atypical in autoimmune encephalitis with neuronal surface antibodies. Compared with other forms of IMCAs, more symptoms of encephalopathy, a higher rate of pleocytosis and positive OB in CSF, and positive therapeutic effect were the key features of anti-NSAbs-associated CAs.

## Introduction

Over the past few decades, with the discovery of anti-NMDAR encephalitis in 2007 (1), an increasing number of specific neuronal surface antibodies (NSAbs) have been discovered, including LGI1-Ab, CASPR2-Ab, AMPA1/2R-Ab, GABAR-A/B-Ab, and so on (2–5). Anti-NSAbs-associated autoimmune encephalitis presents diverse clinical phenotypes, such as seizures, cognitive decline, psychiatric abnormalities, and autonomic dysfunction. In contrast, symptoms of cerebellar ataxia are reported rarely in patients with positive NSAbs (6–8).

Immune-mediated cerebellar ataxias (IMCAs) are one of the most common symptoms of paraneoplastic cerebellar degeneration (PCD) (9). Previous studies have suggested that CAs are atypical in anti-NSAbs-associated autoimmune encephalitis (6, 7, 10). For example, cerebellar ataxia has been reported in a few patients with anti-NMDAR encephalitis (10, 11). Boyko et al. found that about 15% of patients with anti-CASPR2 encephalitis developed cerebellar ataxia followed by the onset of limbic symptoms or Morvan syndrome (12). Although CAs associated with anti-NMDAR and anti-CASPR2 have been studied in a few cases, they have not been studied systematically. In this study, we summarized the clinical features of 14 patients with anti-NSAbs-associated CAs. Moreover, to explore the distinct features of anti-NSAbs-associated CAs, a study of two overlapping cohorts, including patients diagnosed as IMCAs and patients with NSAbs from 2015 to 2020 in our center, was designed and conducted. The present study focused on the clinical characteristics of patients with anti-NSAbs-associated CAs.

## Methods

### Patient Identification

A retrospective investigation was conducted on outpatient and hospitalized cases in the Department of Neurology, Xuanwu Hospital, Capital Medical University from June 2015 to June 2020 to identify potential patients with anti-NSAbs-associated CAs using the keywords *NSAbs* and *IMCAs*. 54 patients with IMCAs and 191 patients positive for NSAbs were enrolled.

**Figure 1:**
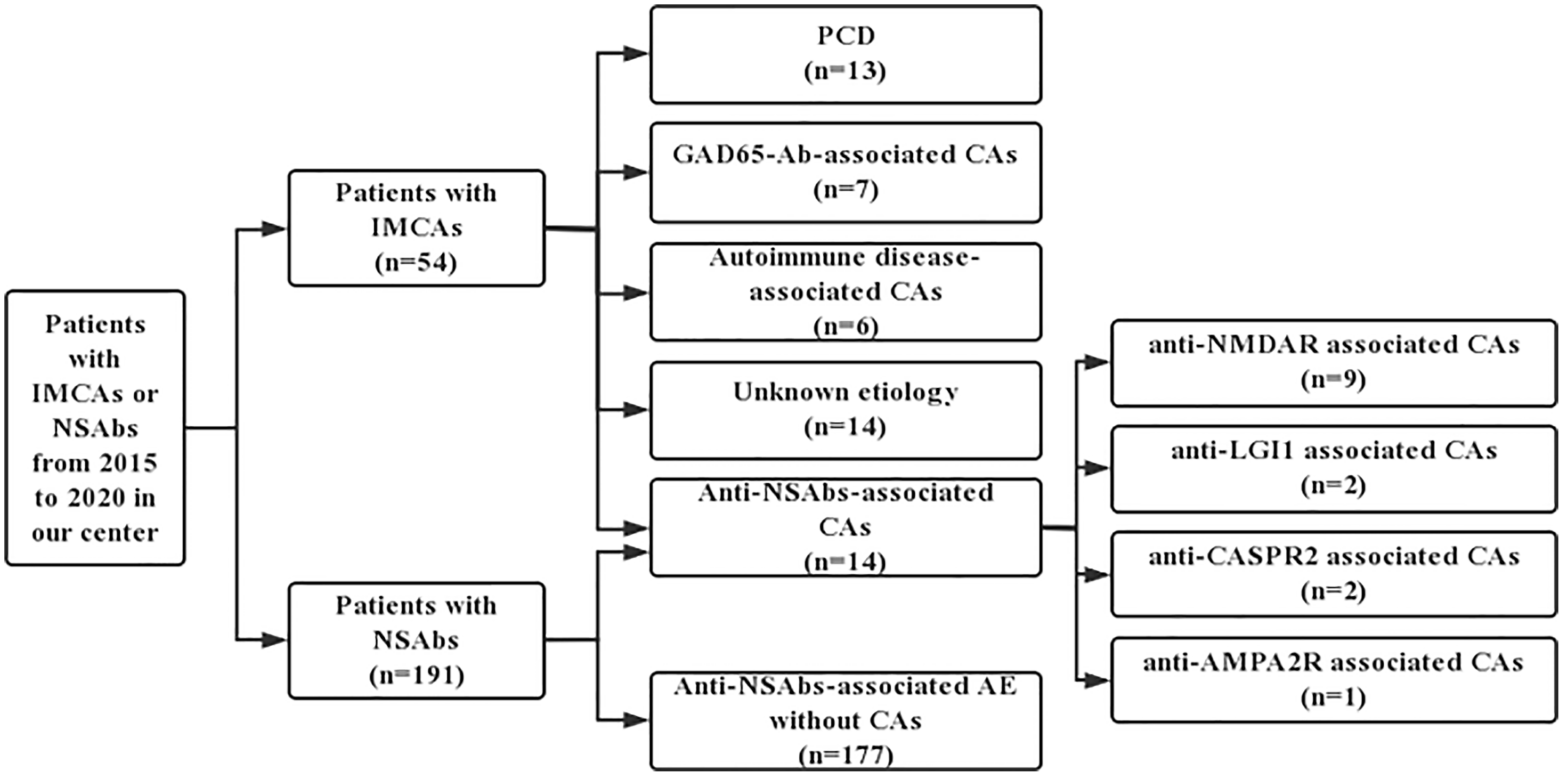
The process of identifying patients from IMCAs and anti-NSAbs cohorts. NSAbs, Neuronal surface antibodies; NSAbs, Neuronal surface antibodies; PCD, Paraneoplastic cerebellar degeneration; AE, autoimmune encephalitis.

All 54 patients were screened for common causes of IMCAs, including PCD, anti-GAD65-Ab-associated CA, post-infectious cerebellitis, gluten ataxia (GA), opsoclonus myoclonus syndrome (OMS), Miller Fisher syndrome, Hashimoto’s encephalopathy (HE) and Systemic Lupus Erythematosus (SLE). Among 54 patients, 13 patients were diagnosed with PCDs (7 with Yo-Ab, 3 with Hu-Ab 2 with Tr-Ab, and 1 with SOX1-Ab), 7 patients with anti-GAD65-Ab-associated CA, 6 with autoimmune disease-associated CAs (4 with Hashimoto’s Encephalopathy and 2 with Systemic Lupus Erythematosus), 14 with unknown etiology and the remaining 14 patients were positive for NSAbs, including 9 with NMDAR-Ab, 2 with LGI1-Ab, 2 with CASPR2-Ab and 1 with AMPA2R-Ab. **Figure 1** demonstrates the process of identifying patients in this study. These 14 patients were negative for onconeural antibodies (ONAs), anti-GAD-65 antibodies, GQ1b antibodies, anti-gliadin antibodies (AGA), anti-thyroid antibodies (ATA), anti-nuclear antibody (ANA), and anti-double-stranded DNA antibodies. In addition, there was no history of virus infection, dermatitis herpetiformis (DH), and celiac disease (CD) in all. Moreover, routine screening examinations, including muti-tumor markers and whole-body PET-CT, showed no malignant tumors in these 14 cases. Alternative causes of cerebellar autoimmunity, such as Gluten Ataxia, PCD, anti-GAD65-Ab-associated CA, and autoimmune disease-associated CA, were excluded.

Patients with PCD, anti-GAD65-Ab-associated CA, and autoimmune disease-associated CAs were included as the control groups to explore the clinical characteristics of IMCAs associated with these antibodies. Then we reviewed the clinical information of the remaining 177 patients with antibodies targeting NSAbs, and all patients met the diagnostic criteria for autoimmune encephalitis (13). We compared the clinical characteristics of patients with or without CAs to identify the occurrence rate of IMCAs in autoimmune encephalitis.

### Antibody Detection

All patients were screened for immunoglobulin G (IgG) against common antigens of autoimmune encephalopathy antibodies using indirect immunofluorescence assays (IFAs) (EUROIMMUN, FA112d-1, Germany) and the cell-based assays Euroimmun kit (commercial CBA) prior to the treatments, including antibodies targeting NMDAR, LGI1, CASPR2, AMPA1/2-R, GABA-A/B-R, DPPX, IgLON5, MOG, and onconeural antibodies (ONAs), including Hu-Ab, Yo-Ab, Ri-Ab, CV2-Ab, PNMA2 (Ma-2/Ta) -Ab, Amphiphysin-Ab, SOX1-Ab, Tr-Ab, and GAD65-Ab. As previously reported (4–6), tissue-based assays (TBAs) using rat brain tissue and CBAs using human embryonic kidney 293 (HEK293) cells were utilized for antibodies detection.

The initial dilution titers of serum and CSF were 1:10 and 1:1, respectively. Antibody titers were defined as three levels. For the antibody titers in serum, 1:10, 1:32 to 1:100, and 1:320 or above were defined as weakly positive, positive, and strongly positive, respectively. In CSF, 1:1, 1:3.2 to 1:10, and 1:32 or above were defined as weakly positive, positive, and strongly positive (14).

### Clinical Data and Outcome Measures

Detailed clinical information including demographic, clinical manifestation, CSF analysis, and brain magnetic resonance imaging (MRI) of all patients was collected. The symptoms of cerebellar ataxia were recorded as gait ataxia, slurred speech, limb dysmetria, and nystagmus. All patients received immunotherapy after diagnosis. Glucocorticoids, intravenous immunoglobulin (IVIG), and plasma exchange were classified as first-line therapy with other immunosuppressants as second-line therapy. The therapeutic regimen and responsiveness to immunotherapy of patients were collected, and the outcome was evaluated by modified Rankin score (mRS) after discharge with a reduction of mRS ≥1 during follow-ups defined as efficacious. Relapse of encephalitis was defined as the new onset or worsening of symptoms occurring after at least 2 months of improvement or stabilization (10).

### Statistical Analysis

Statistical analysis was performed with IBM SPSS V.23.0. Summary statistics were reported as median (range, minimum-maximum) for continuous variables, frequencies, and percentages for categorical variables. As appropriate, clinical data were compared using Pearson’s χ2, Fisher’s exact test, or Mann-Whitney U test. P<0.05 was considered statistically significant.

## Results

### Frequency of Anti-NSAbs-Associated CAs

Among the 40 IMCAs with definite etiology, 14 patients (25.9%) were identified as anti-NSAbs associated CAs, followed by PCD (13 patients, 24.1%), anti-GAD65-Ab-associated CAs (7 patients, 13.0%), and autoimmune disease-associated CAs (6 patients, 11.1%) (**Table 1**). Regarding the 191 patients with positive NSAbs (adult, 164; children, 27), 14 patients (7.3%) developed CAs during the disease period. The result showed a similar proportion of cerebellar ataxia, respectively, in adults (12/164, 7.3%) and children (2/27, 7.4%) (P=1.000) (**Table 2**). Compared with other causes of IMCAs or anti-NSAbs without CAs, no distinct features in demographic data were found in patients with Anti-NSAbs-associated CAs

**Table 1:** Comparison of clinical features of four forms of IMCAs.

Variables (n,%)	Anti-NSAbs-associated CAs (n = 14)	PCD (n = 13)	Anti-GAD65-Ab-associated CAs (n = 7)	Autoimmune disease-associated CAs (n = 6)	P
Male(%)	8 (57.1%)	7 (53.8%)	1(14.3%)	2(33.3%)	0.268
Age(year)	33 (16-66)	56 (37-66)	39(23-68)	40.5(21-56)	0.363
**Cerebellar signs**					
Gait ataxia	7 (50%)	13(100%)	6(85.7%)	5(83.3%)	0.010
Limb dysmetria	5 (35.7%)	11(84.6%)	5(71.4%)	4(66.6%)	0.064
Slurred speech	11 (78.6%)	7(53.8%)	3(42.9%)	4(75.0%)	0.361
Nystagmus	5 (35.7%)	6(46.2%)	4(57.1%)	2(33.3%)	0.797
**CAs symptoms**					
As the initial symptom	3 (21.4%)	12(92.3%)	3(42.9%)	1(16.7%)	<0.01
As the only symptom	2 (14.3%)	8(61.5%)	2(28.6%)	1(16.7%)	0.057
**Other symptoms**					
Seizure onset	11 (78.6%)	0	3(42.9%)	2(33.3%)	<0.01
Memory loss	9 (64.3%)	2(15.4%)	4(57.1%)	5(83.3%)	0.017
Mental abnormalities	9 (64.3%)	2(15.4%)	1(14.3%)	4(66.6%)	0.015
**CSF analysis**					
Pleocytosis	10 (71.4%)	1(7.7%)	2(28.6%)	1(16.7%)	<0.01
Elevated protein	4 (28.6%)	3(23.1%)	2(28.6%)	2(33.3%)	1.000
Positive OB	9 (64.3%)	1(7.7%)	0	0	<0.01
**Cerebellar atrophy on MRI**	1 (7.1%)	4(30.8%)	3(42.9%)	2(33.3%)	0.206
**Outcomes**					
Good	7 (50%)	0	1(14.3%)	2(33.3%)	0.011
Mild	3 (21.4%)	2(15.4%)	2(28.6%)	3(50%)	0.454
Poor	4 (28.6%)	11(84.6%)	4(57.1%)	1(16.7%)	<0.01

IMCAs, Immune-mediated cerebellar ataxias; NSAbs, Neuronal surface antibodies; PCD, Paraneoplastic cerebellar degeneration; CAs, Cerebellar ataxias; OB, Oligoclonal bands; CSF, Cerebrospinal fluid; MRI, Magnetic resonance imaging.

**Table 2:** Comparison of clinical characteristic of patients positive for NASbs with and without CAs.

Variables (n,%)	With CAs (n = 14)	Without CAs (n = 177)	P
Male(%)	8 (57.1%)	102 (57.6%)	1.000
Age(year)	33 (16-66)	24 (6-72)	0.419
**Initial symptoms**			
Seizure onset	3 (21.4%)	73 (41.2%)	0.168
Memory loss	2 (14.3%)	24 (13.6%)	1.000
Mental abnormalities	3 (21.4%)	54 (30.5%)	0.681
**Cerebrospinal fluid analysis**			
Pleocytosis	10 (71.4%)	95 (53.7%)	0.268
Elevated protein	4 (28.6%)	86 (48.6%)	0.174
Elevated IgG	2 (14.3%)	71 (40.1%)	0.084
Positive OB	9 (64.3%)	55 (31.1%)	0.025
**Antibodies detection**			
Positive Ab in serum	9 (64.3%)	168 (94.9%)	<0.01
Positive Ab in CSF	13 (92.9%)	142 (80.2%)	0.419
Signs of encephalitis on brain MRI*	7 (50%)	70 (39.5%)	0.573
**Outcomes**			
Good	7 (50%)	92 (52.0%)	1.000
Mild	3 (21.4%)	39 (22.0%)	1.000
Poor	4 (28.6%)	46 (26.0%)	1.000

NSAbs, Neuronal surface antibodies; CAs, Cerebellar ataxias; OB, Oligoclonal bands; CSF, Cerebrospinal fluid; Ab, Antibody; MRI, Magnetic resonance imaging

*Brain MRI hyperintense signal on T2-weighted fluid-attenuated inversion recovery sequences highly restricted to one or both medial temporal lobes (limbic encephalitis), or in multifocal areas involving grey matter, white matter (13).

According to the data, 185 patients were positive with one antibody (108 with NMDAR-Ab, 52 with LGI1-Ab, 12 with CASPR2-Ab, 4 with AMPA2R-Ab, and 9 with GABAR-B-Ab), and 6 patients were positive with two types of antibodies (4 with NMDAR-Ab and CASPR2-Ab, and 2 with CASPR2-Ab and LGI1-Ab) (**Table 3**). Figures suggested that patients with AMPAR-Ab had the highest occurrence of cerebellar ataxia during the course of the disease (1/4, 25%), followed by CASPR2-Ab (2/12, 16.7%), NMDAR-Ab (9/108, 8.3%), and LGI1-Ab (2/52, 3.8%). However, the symptoms of CAs were not apparent among patients with GABAB-R-Ab (0/9, 0%). Moreover, the occurrence of multiple antibodies was not related to the increased frequency of CAs (0/6, 0%).

**Table 3:** The data of antibodies detection in patients with anti-NSAbs.

Antibodies detection	NMDAR (n = 108)	LGI1 (n = 52)	CASPR2 (n = 12)	GABAR (n = 9)	AMPAR (n = 4)	Two antibodies (n = 6)
**In serum (%)**	8 (7.4%)	18 (34.6%)	6 (50.0%)	3 (33.3%)	1 (25.0%)	0
**In CSF (%)**	9 (8.3%)	1 (1.9%)	1 (8.3%)	0	1 (25.0%)	2 (33.3%)
**Both serum and CSF (%)**	91 (84.3%)	33 (63.5%)	5 (41.7%)	6 (66.7%)	2 (50.0%)	4 (66.7%)

NSAbs, Neuronal surface antibodies; CSF, Cerebrospinal fluid.

### Clinical Characteristics of Patients With CAs

Regarding 14 patients with CAs, three patients suffered from gait ataxia as the initial symptom (two with NMDAR-Ab and one with CASPR2-Ab). The other 11 patients experienced cerebellar ataxia during the first two months after the disease onset (2-60 days, average on day 10). CAs installed acutely (10 patients, 71.4%), subacutely (1 patient, 7.1%) and insidiously (3 patients, 21.4%). Cerebellar signs are shown in **Table 1**. Most patients presented with slurred speech as a main symptom (78.6%), but nystagmus seldom appeared (35.7%). In addition, two patients (one with NMDAR-Ab and the other with CASPR2-Ab) had isolated CAs throughout the disease (**Table 4**). The neurological examination revealed that five patients (35.7%) failed to complete the alternating movement test with both two hands, six patients (42.9%) went through the finger-nose test with difficulties, and five patients (35.7%) could not finish the heel-knee-tibia test. However, there were not enough patients for clinical characteristics comparison among different antibodies.

**Table 4:** Clinical characteristics of patients with anti-NSAbs-associated CAs.

Case	Gender	Age	Antibody	Initial symptom	Cerebellar ataxia			Accompanying symptoms	CSF				MRI		Tumor	Immunotherapy	Follow-up time (months)	Therapeutic effect of cerebellar ataxia
					Onset type	Onset time (day)	Manifestations		WBC	Protein	IgG	OB	Cerebellar hyperintensity	Cerebellar atrophy				
1	M	55	AMPAR	memory decline	acute	4	gait ataxia, slurred speech	seizure onset, mental abnormalities, autonomic dysfunction	33	18	3.51	Pos	Yes	No	No	Steroids, IVIG, plasma exchange and immunosuppressant	24	Poor
2	F	40	CASPR2	limbs numbness	subacute	15	gait ataxia, slurred speech	neuropathic pain, muscle cramps, autonomic dysfunction	6	40	4.76	Pos	Yes	No	No	Steroids	15	poor
3	M	35	CASPR2	cerebellar ataxia	acute	1	gait ataxia, nystagmus	No	0	38	2.19	Neg	No	Yes	No	Steroids	24	Full recovery
4	M	64	LGI1	seizure onset	insidious	30	slurred speech	memory decline	1	25	2.14	Neg	No	No	No	Steroids	42	Relieved significantly
5	M	67	LGI1	confusion	acute	10	gait ataxia	seizure onset, mental abnormalities, autonomic dysfunction	1	51	3.93	Neg	No	No	No	Steroids and IVIG	40	Relieved mildly
6	M	43	NMDAR	mental abnormalities	insidious	30	gait ataxia, slurred speech, nystagmus	seizure onset, memory decline	35	28	3.94	Pos	No	No	No	Steroids and IVIG	36	Poor
7	M	66	NMDAR	mental abnormalities	insidious	60	gait ataxia, slurred speech,limb dysmetria	seizure onset, memory decline	15	33	3.87	Pos	Yes	No	No	Steroids and IVIG	30	Poor
8	M	27	NMDAR	memory decline	acute	2	slurred speech, nystagmus	seizure onset, mental abnormalities	11	36	3.65	Pos	No	No	No	Steroids	15	Relieved significantly
9	F	16	NMDAR	mental abnormalities	acute	5	slurred speech, limb dysmetria	seizure onset, autonomic dysfunction, memory decline	18	29	2.29	Pos	No	No	No	Steroids	16	Relieved significantly
10	F	17	NMDAR	seizure onset	acute	10	slurred speech, limb dysmetria	mental abnormalities, memory decline	8	24	2.13	Neg	No	No	No	Steroids	48	Relieved mildly
11	F	27	NMDAR	cerebellar ataxia	acute	1	Nystagmus, limb dysmetria	seizure onset, mental abnormalities, autonomic dysfunction	89	89	12.6	Pos	No	No	No	Steroids	27	Relieved significantly
12	F	33	NMDAR	seizure onset	acute	10	slurred speech	mental abnormalities, memory decline	1	14	3.55	Pos	No	No	No	Steroids	48	Relieved mildly
13	F	65	NMDAR	cerebellar ataxia	acute	1	gait ataxia, slurred speech, nystagmus	No	2	30	1.64	Neg	Yes	No	No	Steroids	18	Full recovery
14	M	41	NMDAR	confusion	acute	10	slurred speech, limb dysmetria	seizure onset, memory decline	37	32	9.27	Pos	No	No	No	Steroids and IVIG	27	Relieved significantly

NSAbs: Neuronal surface antibodies; CAs: Cerebellar ataxias; M: Male; F: Female; CSF: Cerebrospinal fluid; WBC: White blood cells counts; Pos: Positive; Neg: Negative; OB: Oligoclonal bands; MRI: Magnetic resonance imaging; IVIG: Intravenous immunoglobulin.

### Laboratory Testing and MRI Analysis

Blood and CSF markers indicating inflammation were evaluated. Among patients with NSAbs, oligoclonal bands (OB) in CSF were more frequently in patients with anti-NSAbs-associated CAs than in patients without CAs (P=0.025). The proportions of negative, weakly positive, positive and strongly positive NSAbs were further analyzed (**Table 5**). Interestingly, more patients with CAs were found to have high titer range of CSF antibodies (within “positive” group and “strongly positive” group, 85.7% *vs*. 45.8%, P=0.005). All 14 patients underwent 3T brain MRI. Cerebellar abnormalities on MRI are listed in **Table 4**. Limbic system involvement (medial temporal T2/FLAIR signal changes) (13) was found in seven patients (50%). All 14 cases were negative for routine tumor screening.

**Table 5:** The levels of antibodies titers in patients with anti-NSAbs-associated CAs.

Range of antibody titers	With cerebellar ataxia (n = 14)		Without cerebellar ataxia (n = 177)	
	Serum	CSF	Serum	CSF
**Negative**	5 (35.7%)	1 (7.1%)	9 (5.1%)	35 (19.8%)
**Weakly positive**	1 (7.1%)	1 (7.1%)	54 (30.5%)	47 (26.6%)
**Positive**	8 (57.1%)	7 (50%)	89 (50.3%)	50 (28.2%)
**Strongly positive**	0	5 (35.7%)	11 (6.2%)	31 (17.5%)

NSAbs, Neuronal surface antibodies; CAs, Cerebellar ataxias; CSF, Cerebrospinal fluid.

### Response to the Immunotherapy

All 14 patients were followed up for at least 15 months after discharge (median 27 months, ranging from 15-48 months). At the early stage, the patients were investigated by the standardized questionnaire through a telephone survey once a month, and all patients were hospitalized again for disease re-evaluation 6 months after discharge. Two patients were free from the symptoms of cerebellar ataxia, and eight recovered satisfactorily (10/14, 71.4%) after the glucocorticoid treatment or the combination of glucocorticoids and IVIG (**Table 4**). In addition, two patients (patients 8 and 12) with NMDAR-Ab had clinical relapses, whereas the symptoms of cerebellar ataxia were not observed during the relapses. No malignancy was found in these 14 patients until the last follow-up visit. Follow-up brain MRI showed an obvious diminishment in cerebellar T2/FLAIR-hyperintense lesions in two patients (2/4, 50%, case 7 and 13) and no significant changes in others (case 1 and case 2). However, cerebellar atrophy in brain MRI of case 3 remained unchanged.

## Discussion

IMCAs are a rare spectrum of diseases, and several specific neuronal antibodies were identified in IMCAs, such as the onconeural antibodies (Yo-Ab, Hu-Ab, CV2-Ab, Ri-Ab, and Ma-2-Ab) for PCDs, GAD65-Ab for anti-GAD65 Ab-associated CA, and Ri-Ab for OMS (15–20). However, only a few studies have concentrated on the anti-NSAbs-associated CAs in autoimmune encephalitis (6–8). Research conducted by Iizuka T et al. revealed that approximately 5% of patients with anti-NMDAR encephalitis showed cerebellar complaints during the disease course (11). Boyko et al. reviewed the clinical data of 163 patients with CASPR2-Ab and found that 24 patients (14.7%) developed CAs (12). However, no large-sample clinical observation and follow-up studies have been conducted to explore the frequency of cerebellar ataxia in patients with anti-AMPAR, anti-LGI1, or anti-GABABR encephalitis by far (8, 21).

This study reviewed 54 patients with IMCAs and identified 14 patients carrying different types of neural surface autoantibodies, including NMDAR-Ab, LGI-Ab, CASPR2-Ab, and AMPA2R-Ab. Anti-NSAbs-associated CAs mostly appeared in the first two weeks of the disease course of autoimmune encephalitis, and two patients (case 3 with CASPR2-Ab and case 13 with NMDAR-Ab) had isolated symptoms of CAs throughout the disease. Five patients with isolated CAs have been reported from publications previously (7, 22), including four with CASPR2-Ab and one with NMDAR-Ab. Although the symptoms of isolated CAs were common in PCD, when patients suffered from isolated CAs, the diagnosis of autoimmune encephalitis should also be considered, especially anti-NMDAR or anti-CASPR2 encephalitis. Anti-NSAbs-associated CAs showed less predominant truncal ataxia than PCDs. Mimicking Hashimoto’s encephalopathy (23, 24), anti-NSAbs associated more frequently with additional extra-cerebellar symptoms, such as seizures and mental abnormalities, during the disease course.

Compared with other forms of IMCAs, a higher rate of pleocytosis and positive OB in CSF might have a predictive value for cerebellar symptoms in autoimmune encephalitis. In terms of imaging abnormalities on MRI, previous studies figured out that only 6% patients with anti-NMDAR encephalitis had cerebellar abnormalities and 13.3% patients suffered from progressive and irreversible cerebellar atrophy (11, 25). However, cerebellar atrophy in MRI was found in 16.7% patients with CASPR2-Ab (26) and those patients all benefited from immunotherapy (26). In this study, only one patient (case 3) with CASPR2-Ab had cerebellar atrophy. After immunotherapy, he was free from symptoms, and the existing cerebellar atrophy did not worsen in follow-up brain MRI.

All 14 patients received immunotherapy, and most encouragingly, two patients with isolated CAs (case 3 mentioned above and case 13) fully recovered and returned to work. Five patients presented with isolated CAs as reported previously, including 4 patients with CASPR2-Ab and 1 with NMDAR-Ab mentioned above (7, 22). The latter patient exhibited a partial improvement after immunotherapy (22), while the therapeutic response of the former 4 patients is unknown (7). The influencing factors of treatment effect were unclear, which might be related to the type of antibodies, the influence of accompanying symptoms and the severity of cerebellar inflammation. Further research is needed to explore the underlying mechanism of the pathophysiological function and the factors affecting the immunotherapy of anti-NSAbs associated CAs.

Among the many mechanistic studies of IMCAs, the association between ONA and PCDs has always been a research hotspot, but few studies have focused on the pathogenesis of anti-NSAbs associated CAs. In patients with PCD, previous studies have revealed a significant loss of Purkinje cells due to a cell-mediated cytotoxic immune response associated with activated CD8+ T cells and microglia in the cerebellum (27, 28). However, in patients with anti-NMDAR encephalitis, post-mortem studies demonstrated no profound loss of Purkinje cells in the cerebellum (11). The NMDAR is strongly expressed on the cerebellum and these receptors on granular cells (but not on Purkinje cells) were identified as a specific antigen of IgG antibodies, which might interfere with the excitatory pathway involving the NMDAR-mediated signaling, resulting in cerebellar dysfunction (29).

## Conclusion

Cerebellar ataxias were rare and atypical in autoimmune encephalitis with neuronal surface antibodies. Patients with anti-NSAbs-associated CAs exhibited a higher positivity rate of OB in CSF. Most patients, especially those with isolated CAs, responded well to immunotherapy. Compared with other causes, anti-NSAbs-associated CAs led to more symptoms of encephalopathy and showed better therapeutic effects from immunotherapy. Considering the treatability of anti-NSAbs-associated CAs, it is valuable to perform serum and CSF NSAbs tests for patients with cerebellar ataxia.

## Data Availability Statement

The original contributions presented in the study are included in the article/supplementary material. Further inquiries can be directed to the corresponding author.

## Ethics Statement

The studies involving human participants were reviewed and approved by The Ethics Committee of Xuanwu Hospital (No.2017YFC0907702). The patients/participants provided their written informed consent to participate in this study.

## Author Contributions

YJ was the major contributors in writing the manuscript. YJ, ML, and DL conceptualized the study. YJ, MZ, and HW collected samples and data. ZH, LJ, JY, AL, and YW contributed to the diagnosis and treatment of patients. YW checked the final manuscript. All authors contributed to the article and approved the submitted version.

## Funding

This work was supported by Beijing Postdoctoral Research Foundation [Grant No. 2021-ZZ-001], Beijing Municipal Education Commission [Grant No. TJSH20161002502], National Natural Science Foundation of China [Grant No. 81771398], Beijing Key Clinical Speciality Excellence Project and National Support Provincial Major Disease Medical Services and Social Capability Enhancement Project.

## Conflict of Interest

The authors declare that the research was conducted in the absence of any commercial or financial relationships that could be construed as a potential conflict of interest.

## Publisher’s Note

All claims expressed in this article are solely those of the authors and do not necessarily represent those of their affiliated organizations, or those of the publisher, the editors and the reviewers. Any product that may be evaluated in this article, or claim that may be made by its manufacturer, is not guaranteed or endorsed by the publisher.

## Abbreviations

NSAbs, Neuronal surface antibodies; PCD, Paraneoplastic cerebellar degeneration; ONAs, Onconeural antibodies; MRI, Magnetic resonance imaging; mRS, Modified Rankin score; WBC, White blood cell counts; OB, Oligoclonal bands; IMCAs, Immune-mediated cerebellar ataxias; GA, Gluten ataxia; OMS, Opsoclonus myoclonus syndrome.
